# P-2022. Variability in Peri-operative Antimicrobial Prescribing Practices in the Liver Transplant Setting across the USA

**DOI:** 10.1093/ofid/ofaf695.2186

**Published:** 2026-01-11

**Authors:** Elizabeth Bell, Hannah Imlay, Jennifer Pisano, Jonathan Hand, Alan Koff, Risa Fuller, Margaret Jorgenson, Michael P Angarone, Rebecca Nirmal Kumar, Alfred Luk, Ralph Tayyar, Margaret E McCort

**Affiliations:** University of Chicago, Chicago, IL; University of Utah Health, Salt Lake City, UT; University of Chicago Hospital, Chicago, Illinois; Ochsner Health, New Orleans, LA; UC Davis School of Medicine; Icahn School of Medicine at Mount Sinai, New York, NY; University of Wisconsin, Madison, Wisconsin; Northwestern University Feinberg School of Medicine, Chicago, IL; Georgetown University School of Medicine, Washington , DC; Tulane University, New Orleans, Louisiana; University of Washington, Seattle, Washington; Montefiore Medical Center / Albert Einstein College of Medicine, Bronx, New York

## Abstract

**Background:**

While expert guidelines provide recommendations for peri-operative antimicrobial choice and duration for liver transplant recipients, few randomized studies have been conducted in this population. We aimed to understand transplant center practices around perioperative orthotopic liver transplant (OLT) prophylaxis.

**FIGURE 1:**
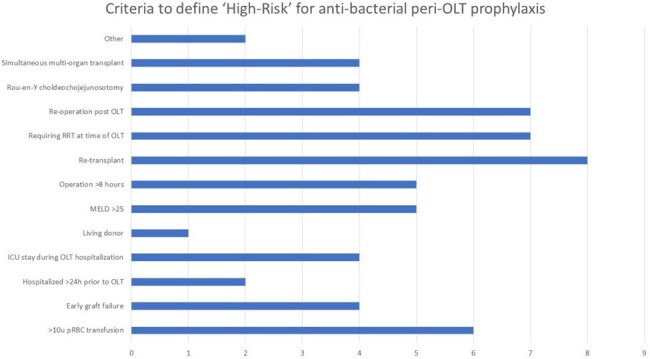
Criteria used to define ‘high-risk’ for antibacterial perioperative OLT prophylaxis in centers who risk stratify antibacterial prophylaxis, n=11 centers. Note that the majority of centers, 72%, required only one criteria to be met to be considered ‘high-risk,’ while the rest required multiple criteria.

**FIGURE 2:**
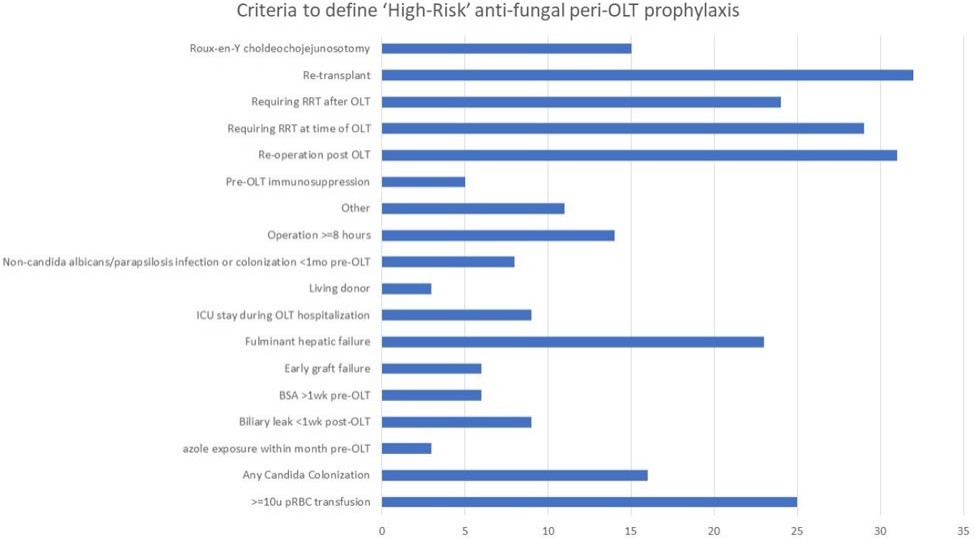
Criteria used to define ‘high-risk’ for antifungal perioperative OLT prophylaxis in centers who risk stratify antifungal prophylaxis, n=30 centers. Note that the majority of centers, 70%, required only one criteria to be met to be considered ‘high-risk,’ while the rest required multiple.

**Methods:**

We sent a survey via REDCap to providers and pharmacists within the American Society of Transplantation’s Infectious Disease Community of Practice discussion board. The survey included questions on institutional practices of peri-OLT antimicrobial choice and duration. Narrow-spectrum antibiotics were defined as lacking anti-pseudomonal coverage such as cefazolin or ceftriaxone with metronidazole or ampicillin-sulbactam. Vancomycin and antifungals were considered separately. Centers were considered to use ‘risk stratification’ if different recommendations for antimicrobial choice or duration were given based upon perceived risk of the recipient.

**FIGURE 3:**
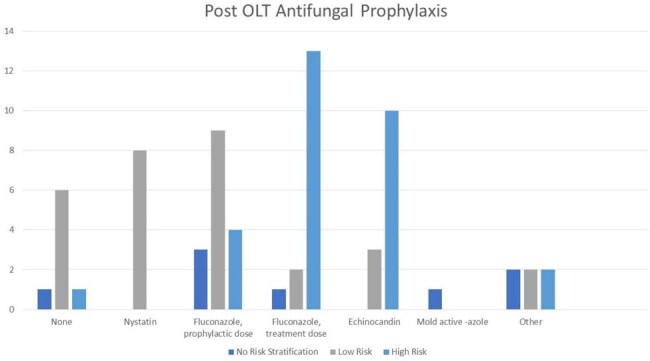
Post OLT antifungal prophylaxis recommendations from centers who do not risk-stratify (dark blue) compared to those who stratified into low-risk (grey) and high-risk (light blue).

**Results:**

Clinicians from 38 unique institutions, representing all 11 UNOS regions, filled out the survey. Most respondents (86%) were physicians, *Table 1*. Few centers (29%) risk-stratified surgical-site peri-OLT antibacterial prophylaxis, while most (79%) used risk-stratification for antifungal prophylaxis. Definitions for ‘high-risk’ were widely variable across both categories, *Figures 1 & 2*, though the most agreed upon criteria included re-transplantation, re-operation after OLT, and requiring renal replacement therapy. While most recommended broad-spectrum antibacterials for less than or equal to 48 hours, there was significantly more variation in both antifungal of choice, *Figure 3*, as well as duration. Few (21%) institutions incorporate microbiologic screening for MRSA, CRE, VRE, and ESBLs into peri-OLT prophylaxis recommendations. Eight centers (21%) had a standardized protocol for recommended antimicrobial use for pre-OLT candidates hospitalized for decompensated liver failure.

**Conclusion:**

There is significant variability between transplant centers in antimicrobial use around the time of OLT. Further research is also needed to determine whether high versus low-risk patients warrant different prophylaxis practices.

**Disclosures:**

Jonathan Hand, MD, AstraZeneca: Advisor/Consultant|AstraZeneca: Grant/Research Support|Ferring: Grant/Research Support|Innoviva: Advisor/Consultant|Janssen: Grant/Research Support|Pfizer: Advisor/Consultant|Pfizer: Grant/Research Support|Scynexis: Grant/Research Support|The Antibiotic Resistance Leadership Group (ARLG): Grant/Research Support|The Antibiotic Resistance Leadership Group (ARLG): Honoraria Margaret Jorgenson, PharmD, BCTXP, Merck: Advisor/Consultant|Merck: Grant/Research Support Rebecca Nirmal Kumar, MD, AstraZeneca: Advisor/Consultant|AstraZeneca: Grant/Research Support|Pfizer: Grant/Research Support

